# A high-throughput real-time *in vitro* assay using mitochondrial targeted roGFP for screening of drugs targeting mitochondria

**DOI:** 10.1016/j.redox.2018.10.013

**Published:** 2018-10-24

**Authors:** Aneesh Chandrasekharan, Shankara Narayanan Varadarajan, Asha Lekshmi, Santhik Subhasingh Lupitha, Pramod Darvin, Leena Chandrasekhar, Prakash Rajappan Pillai, T.R. Santhoshkumar, M. Radhakrishna Pillai

**Affiliations:** Cancer Research Program, Rajiv Gandhi Centre for Biotechnology, Poojappura, Thycaud P.O., Thiruvananthapuram, Kerala 695014, India

**Keywords:** mt-roGFP, mitochondrial targeted reduction-oxidation sensitive Green Fluorescent Protein2, H2B-mCherry, Histone 2B-mCherry, IMS-RP, Inter-Membrane Space Reporter Protein, rxYFP, redox-sensitive Yellow Fluorescent Protein, EGFP, Enhanced Green Fluorescent Protein, TMRM, Tetramethyl-rhodamine Methyl Ester, EGCG, EpiGalloCatechin Gallate, 17AAG, 17-(Allylamino)-17-demethoxygeldanamycin, DMEM, Dulbecco's Modified Eagle Medium, RPMI 1640, Roswell Park Memorial Institute 1640, HTS, High-Throughput Screening, FRET, Förster Resonance Energy Transfer, NA, Numerical Aperture, HyPer-red, Red fluorescence-based hydrogen peroxide sensor, Smac, Second mitochondria-derived activator of caspases, Mitochondria, Mitochondrial oxidation, roGFP, Apoptosis, Drug screening

## Abstract

Most toxic compounds including cancer drugs target mitochondria culminating in its permeabilization. Cancer drug-screening and toxicological testing of compounds require cost-effective and sensitive high-throughput methods to detect mitochondrial damage. Real-time methods for detection of mitochondrial damage are less toxic, allow kinetic measurements with good spatial resolution and are preferred over end-stage assays.

Cancer cell lines stably expressing genetically encoded mitochondrial-targeted redox-GFP2 (mt-roGFP) were developed and validated for its suitability as a mitochondrial damage sensor. Diverse imaging platforms and flow-cytometry were utilized for ratiometric analysis of redox changes with known toxic and cancer drugs. Key events of cell death and mitochondrial damage were studied at single-cell level coupled with mt-roGFP. Cells stably expressing mt-roGFP and H2B-mCherry were developed for high-throughput screening (HTS) application.

Most cancer drugs while inducing mitochondrial permeabilization trigger mitochondrial-oxidation that can be detected at single-cell level with mt-roGFP. The image-based assay using mt-roGFP outperformed other quantitative methods of apoptosis in ease of screening. Incorporation of H2B-mCherry ensures accurate and complete automated segmentation with excellent Z value. The results substantiate that most cancer drugs and known plant-derived antioxidants trigger cell-death through mitochondrial redox alterations with pronounced ratio change in the mt-roGFP probe.

Real-time analysis of mitochondrial oxidation and mitochondrial permeabilization reveal a biphasic ratio change in dying cells, with an initial redox surge before mitochondrial permeabilization followed by a drastic increase in ratio after complete mitochondrial permeabilization. Overall, the results prove that mitochondrial oxidation is a reliable indicator of mitochondrial damage, which can be readily determined in live cells using mt-roGFP employing diverse imaging techniques. The assay described is highly sensitive, easy to adapt to HTS platforms and is a valuable resource for identifying cytotoxic agents that target mitochondria and also for dissecting cell signaling events relevant to redox biology.

## Highlights

• Mitochondrial oxidation is an universal marker for mitochondrial damage and mitochondrial permeabilization.
• Ratiometric imaging of mt-roGFP in high-throughput mode allows rapid screening of compounds that target mitochondria.
• Real-time ratiometric imaging of mt-roGFP and mitochondrial permeabilization reveals a biphasic redox alteration in cells undergoing mitochondrial permeabilization.

## 1 Introduction

The currently used cancer drugs are diverse in structure, activity and target specificity. Despite this varied and complex target specificity and target availability in different cancer types, most drugs are expected to kill the cancer cells through apoptosis or confer cytostatic activity. This is the rationale for employing cytotoxicity as a preliminary screening strategy for cancer drug discovery by the successful NCI developmental therapeutics program. The NCI approach and the COMPARE program are currently the best *in vitro* cytotoxic models because of their ability to predict the mechanism of action of the drugs to some extent [1]. DNA damage, proteotoxic stress, mitochondrial damage, and redox alterations contribute to cell toxicity. Among them, mitochondrial damage and DNA damage have been extensively used for cancer drug screening and toxicological evaluation of environmental toxicants [2], [3], [4], [5], [6], [7].

As mitochondria are involved in all metabolic processes and ATP production needed for performing diverse physiological functions, mitochondrial damage often underlies various pathologies. Most known toxicants exert their activity through its impact on mitochondrial functions. Mitochondrial membrane potential, ATP assay, oxygen consumption, and extracellular flux analysis have been employed as a measure of mitochondrial damage while profiling toxicity of chemical compounds [8], [9], [10]. Although high-throughput adaptable, most of these methods require expensive reagents and are laborious to perform. Real-time capabilities of such methods are also limited.

Many innovative approaches were ascertained in developing Green fluorescent protein (GFP) based sensors for signaling molecules such as calcium, reactive oxygen species (ROS), ATP, pH, cAMP [11], [12], [13], [14], [15]. Genetically encoded fluorescent probe based cell death tools were also described for toxicological studies [16], [17], [18], [19]. We have described a high-throughput FRET-based method for caspase profiling [16], [20]. Intracellular generation of ROS and dysfunctional redox signaling have been attributed to the cytotoxic activity of various antitumor agents. The popular chemotherapeutics such as cisplatin, paclitaxel, bortezomib, etoposide and target-specific agents such as sorafenib, 17AAG, Gleevec are reported to exert strong oxidizing activity on cells and organelles [21], [22], [23], [24]. Although oxidative stress is a primary contributing factor for drug activity, redox-based real-time drug screening tools are yet to be explored. The currently available chemical probes of redox sensors are not specific and generate toxicity. The genetically encoded GFP based reduction-oxidation sensitive probe roGFP respond to the glutathione redox potential with dual excitation property [25]. The approach subsequently allowed to visualize the dynamics of thiol-based redox alterations in diverse physiological and pathological conditions and multiple organelles within cells [25], [26]. Several other spectrally varying genetically encoded probes were also reported for glutathione and hydrogen peroxide detection [27], [28].

Here, we describe the development of stable cells expressing roGFP targeted at mitochondria (mt-roGFP) and nuclear H2B-mCherry [29], a tool for real-time imaging of mitochondrial oxidation as an approach for screening cytotoxic compounds in a high-throughput manner. The study shows that mitochondrial oxidation is an universal marker for mitochondrial permeabilization. This event can be detected at single-cell level using redox sensing GFP with high spatiotemporal resolution and potential utility in high-throughput screening (HTS). Simultaneous imaging of mitochondrial redox alterations and mitochondrial permeabilization in dying cells reveal that mitochondrial oxidation is an initiator of mitochondrial permeabilization and mitochondria undergo rapid secondary oxidation upon completion of mitochondrial permeabilization. The method described here will allow addressing crucial fundamental signaling involved in redox changes and mitochondrial permeabilization in ascertaining compound activity profiling.

## 2 Materials and methods

### 2.1 Cell culture and transfection

Osteosarcoma cell line U2OS was procured from ATCC (Manassas, VA, USA) and used within seven passages of revival. Ovarian cancer cell line OVCAR8 was obtained from NCI (Rockville, MD, USA) and employed within ten passages after revival. Cells were maintained in DMEM/High glucose (HyClone, S.Logan, UT, USA) containing 10% Fetal Bovine Serum (HyClone, S.Logan, UT, USA) and 1% antibiotics (antibiotic-antimycotic cocktail, Invitrogen, Carlsbad, CA, USA) in a humidified CO_2_ (5%) chamber at 37 °C. The cells were seeded in 96 well optical bottom plates for high-resolution imaging experiments. At 50–70% confluency, the medium was removed, and 100 µl of the compounds to be tested prepared in fresh phenol-red free DMEM (Invitrogen, Carlsbad, CA) containing 5% FBS was added. The list of drugs with respective concentration used for the current study is given in Supplemental information (Table S1).

The expression vectors Bax-EGFP and Cytochrome *c*-EGFP were described previously [22]. The expression vector used for live-cell mitochondrial permeabilization pBabe-puro-IMS-RP was a gift from Peter Sorger (Addgene plasmid # 24535) [30]. Mitochondrial-targeted roGFP2 (mt-roGFP) was procured from Prof. S. James Remington (Institute of Molecular Biology, University of Oregon, Eugene, OR). The expression vector H2B-mCherry was a gift from Robert Benezra (Addgene plasmid # 20972). The expression vector pcDNA3- Smac-mCherry was a gift from Douglas Green (Addgene plasmid # 40880). The expression vector HyPer Red targeted to the mitochondrial matrix was a gift from Dr. Vsevolod V Belousov. MitoQ was a gift from Dr. William Stow, MitoQ Ltd. Cell transfection was performed using Lipofectamine® LTX with Plus™ Reagent (Invitrogen, Carlsbad, CA, USA) as per the manufacturer's protocol. Generation of stably expressing clones was by antibiotic selection of the cells in 800 µg/ml of G418 (Invitrogen, Carlsbad, CA, USA) containing medium for 30–45 days. Cells with different levels of transgene expression were clonally expanded and sorted through a tight gate to obtain clones with a homogeneous level of expression by FACSAria III (Becton Dickinson, San Jose, CA, USA). For sorting of roGFP cells, emission signal at 520/40 nm was collected while exciting with 405 nm and 488 nm laser lines in 405/488 ratio mode. For the transfection of IMS-RP, cells were electroporated using Neon transfection (Invitrogen, Carlsbad, CA, USA) system as per the manufacturer's protocol. For generating stable cells expressing both mt-roGFP and H2B-mCherry, stable cells expressing mt-roGFP were further transfected with H2B-mCherry followed by flow-cytometric sorting to enrich double transfected population. Further single cell clones were expanded and used for the current study.

### 2.2 Well plate imaging using Confocal laser scanning microscopy

Cells seeded in 96-well optical bottom plates were sequentially scanned using a laser scanning Leica TCS SP8 Confocal microscope (Leica Microsystems, Wetzlar, Germany) equipped with supercontinuum white light laser. 405 diode laser line was coupled with a 488 nm and a 562 nm line from white light for sequential imaging. The emission at 515 ± 15 nm was collected while exciting at 405 and 488 nm using GaAsP detectors in ratio mode. For TMRM, the emission at 600 ± 25 was collected while exciting at 562 nm. For live imaging, the cells were maintained at 37 °C at optimum humidity and 5% CO_2_ in a microscope incubation chamber (Leica Microsystems, Wetzlar, Germany).

### 2.3 Fluorescence and time-lapse imaging

Cells were grown in a chambered coverglass (Greiner Bio-One, Austria), to which, the indicated drugs reconstituted in phenol-red free DMEM containing 5% FBS was added. Cells were stained as described and positioned in a live-cell incubation chamber (Tokai Hit, Japan) that maintains optimum CO_2_, temperature, and humidity. Imaging was carried out with a 20× Plan Apo 0.7NA objective under an inverted fluorescence microscope (Nikon Eclipse, TiE). The images were acquired using EMCCD camera Andor iXON 885 using NIS elements (Nikon Instruments Inc. USA) at defined intervals for the indicated time period. Photobleaching was curtailed by reducing the intensity of the light to 1% by intensity iris control. For ratio imaging of roGFP two excitation filters, 400/15 nm and 480/30 nm placed in a filter wheel were alternated while collecting the emission at 535/25 nm using the dichroic 505 nm LP. For Annexin V staining with redox alterations, the cells were stained with Alexa Fluor 647 conjugated Annexin V (Molecular Probes) as per the manufacturer's instruction. The Annexin V was detected using a filter set of CY7. Imaging of Bax-EGFP was carried out using 480/30 nm placed in an excitation filter wheel, while collecting the emission at 535/25 nm using the dichroic 505 nm LP, IMS-RP, Smac mCherry, and H2B mCherry image acquisition was through a filter combination of Ex: 545 ± 30 nm, Em: 620 ± 60 nm and dichroic 570 nm LP. Segmentation and post-acquisition analysis was carried out using Nikon NIS-elements 4.0 software.

### 2.4 Detection of roGFP by flow cytometry

roGFP ratio analysis by FACS was performed in a FACSAria III (BD Biosciences) equipped with a 405 nm and 488 nm laser lines. Briefly, cells seeded in 12-well plates were treated with drugs. Post drug treatment, the cells were trypsinized, washed twice with PBS and passed through a 40 µm sieve (BD Biosciences) before analysis. The cells were excited with 405 nm, and 488 nm lasers and fluorescence emission was collected at 520/40 nm at each laser line in ratio mode. For mitochondrial membrane potential analysis using TMRM, the red emission was collected using 620/40 filter sets by exciting with 562 nm laser line.

### 2.5 Live-cell staining

Cells were counterstained with TMRM (Molecular Probes #T-668) or MitoSOX™ Red (Molecular Probes #M36008) as per the standard protocol. For TMRM staining, cells were incubated with 100 nM TMRM at 37 °C for 10 min following which the unbound dye was washed off. Stained cells were replenished with 5 nM TMRM containing 5% phenol red-free RPMI for imaging mitochondrial membrane potential loss. MitoSOX™ Red mitochondrial superoxide indicator is a fluorogenic dye for highly selective detection of superoxide in the mitochondria of live cells. Cells were stained with 5 µM MitoSOX™ Red in 5% RPMI and incubated in the dark for 10 min at 37 °C. Cells were gently washed with PBS to remove excess unbound dye and replaced with fresh 5% phenol-red free RPMI.

### 2.6 High-throughput screening: acquisition, segmentation, and analysis

For high-throughput imaging, cells were seeded in 96-well optical bottom plates (Greiner Bio-One, GmbH). After attaining 60–70% confluency, the medium was removed and replenished with phenol-red free DMEM containing 5% FBS with the compounds to be tested at the appropriate working concentrations. Plates were imaged with BD Pathway™ 435 Bioimager (Becton Dickinson Biosciences, USA). The filter combination for roGFP was designed as described for microscopic imaging with independent automated control of excitation, emission and dichroic filter wheels for easy adaptation of roGFP ratio image acquisition with other parameters of choice. For roGFP ratio imaging two excitation filters 405/12 nm and 488/28 nm were placed in the excitation wheel, emission at 520/40 was collected through the dichroic 405 nm LP. For H2B mCherry, 562/40 nm excitation and emission at 620/40 nm were collected as described. Images for each well were acquired in the respective channels using a dry 20X objective with NA 0.75. As the cells have a high and homogeneous expression, an average exposure time of 100–200 ms is sufficient to acquire the images. Hence, a high-throughput screening in 96-well plate can be performed within 15 min time in 2 × 2 montage.

### 2.7 Post-acquisition segmentation and quantification

Post-acquisition analysis of multiwell imaging was done using BD AttoVision™ (Version 1.6/435, Becton Dickinson Biosciences, USA) software. For proper automated segmentation of cells, nonspecific background noise was subtracted using background reference image. The intensity-based automated thresholding was done for identifying and separating cells expressing mt-roGFP using polygon tool to generate output ROIs for further analysis. For segmentation of cells expressing nuclear H2B, polygon ring based segmentation to identify a perinuclear region on the roGFP channel was performed through dilation using a nuclear polygon band on the red channel. For spatial distinction of cytoplasmic and nuclear regions the automatic threshold level, dilation width, as well as erosion factor, were optimized. After proper segmentation of all cells across the wells, required measurement features such as intensity, granularity, average intensity, area, and ratio were opted for all the fluorescent channels and applied to all the wells to generate high-content data. Data output was in the form of images and dot plots, with ratio changes (405/488) corresponding to TMRM intensity loss, and MitoSOX Red intensity. Data were expressed as average ± standard deviation as indicated. The inbuilt algorithm within AttoVision software was employed for calculating Z′ factor using cells treated with a standard concentration of the drugs to generate a maximum response and mock-treated wells as a negative control. For correlation of transmembrane potential and mt-roGFP ratio change, all the cells in a representative field were segmented and analyzed for TMRM fluorescence intensity vs. ratio. The ROI readouts were exported to GraphPad prism for calculating correlation index. Biological experiments were performed in triplicate, and statistical analysis was performed using a two-way analysis of variance (GraphPad Prism). *p* < 0.05 was considered to be significant.

## 3 Results

### 3.1 Mitochondrial ROS generation correlates with Bax activation and Cytochrome *c* release

Cells stably expressing cytochrome *c*-EGFP have been used as a marker for mitochondrial permeabilization and apoptosis detection in image-based drug screening platforms [31], [32]. To study the correlation of cytochrome *c* release and mitochondrial ROS, cytochrome *c*-EGFP stable cells stained with mitochondrial ROS specific dye MitoSOX red was employed. Upon drug treatment, only cells with increased ROS as indicated by MitoSOX red fluorescence showed cytochrome *c* release, while none of the cells with intact mitochondria showed MitoSOX red fluorescence (Supplementary Fig. 1).

The early events that contribute to mitochondrial permeabilization are activation of Bax and its translocation to mitochondria. Cancer cells expressing Bax-EGFP fusion protein were used to analyze the Bax activation and its translocation in live cells. Induction of apoptosis in cells expressing Bax-EGFP also confirmed that mitochondrial translocation of Bax corresponds with an increase in mitochondrial ROS (Supplementary Fig. 2). Taken together, the above results confirm that the increase in mitochondrial ROS is a reliable marker for mitochondrial damage and its permeabilization.

**Fig. 1:**
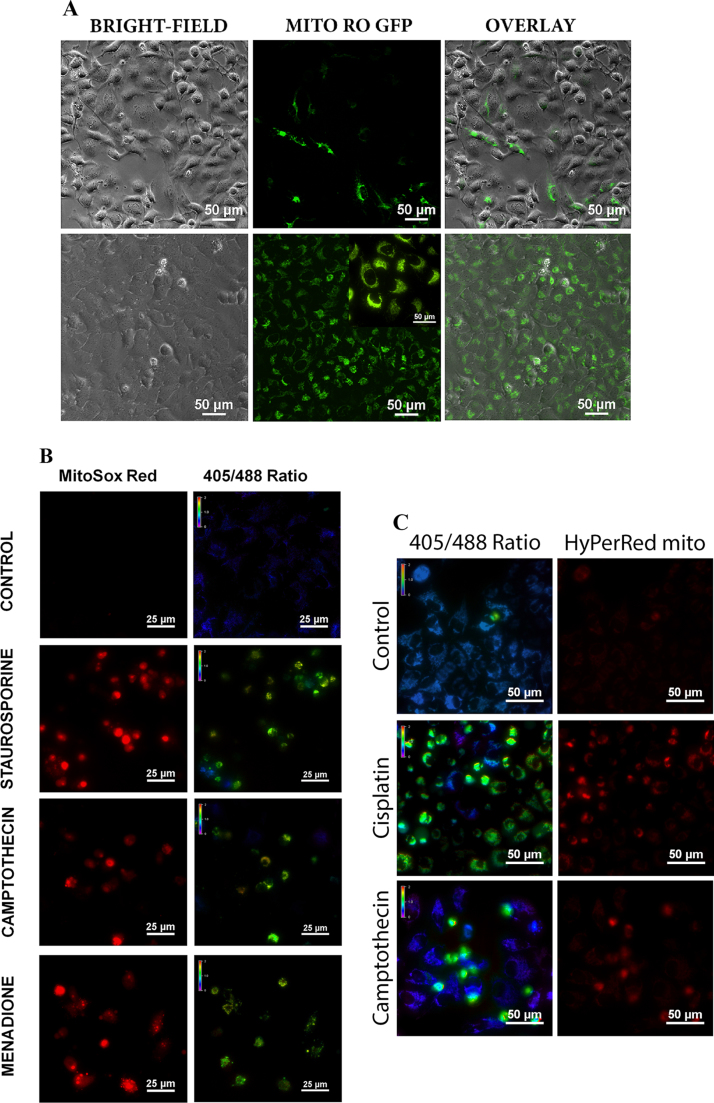
(A). U2OS cells were transfected with roGFP targeted at mitochondria as described. The bright field and respective EGFP channels are shown. The above panel shows heterogenous expression of roGFP. The below panel represents a single cell colony expanded after selection that shows the homogenous level of expression. The inset shows an enlarged part of the image. (B). U2OS cells stably expressing mt-roGFP were left untreated or treated with staurosporine (2 µM), camptothecin (30 µM), Menadione (20 µM) for 24 h. The cells were stained with MitoSOX Red to detect Mitochondrial ROS as described. Control cells showed no MitoSOX Red signal with an average ratio of 0.5 for roGFP. In treated cells, there is a significant increase in MitoSOX Red fluorescence concomitant with an increase in roGFP ratio. (C). U2OS cells stably expressing mt-roGFP and HyPer-red targeted at mitochondria were left untreated or treated with cisplatin (150 µM), camptothecin (30 µM) for 24 h. Control cells showed low HyPer-Red signal with an average ratio of 0.5 for roGFP. In treated cells, there is a significant increase in HyPer-Red fluorescence corresponding with an increase in roGFP ratio.

To visualize mitochondrial oxidation, a real-time live cell tool was generated by transfecting cancer cells with mt-roGFP. This probe changes excitation maxima from 488 nm to 405 nm upon oxidation and enables ratiometric detection of mitochondrial oxidation. A homogeneous level of expression is essential for proper and complete automated segmentation. Colony selection was employed to get the appropriately targeted cells with similar expression (Fig. 1A and Supplementary Fig. 3). The probe showed complete localization to mitochondria indicating its specific organelle targeting. MitoROS intensity with MitoSOX staining correlated with the redox ratio changes confirming that roGFP probe in the ratio mode is a real reflection of apoptotic mitochondrial damage (Fig. 1B). An intensity-based genetically encoded red fluorescent indicator of Hydrogen peroxide, HyPer targeted at mitochondrial matrix was also employed to analyze the correlation of roGFP ratio change and hydrogen peroxide at Mitochondria [33]. The cells with increased roGFP ratio also showed increased HyPer Red signal in drug-treated cells demonstrating close correlation with HyPer Red signal and roGFP ratio (Fig. 1C).

### 3.2 Mitochondrial ROS status corresponds with mitochondrial transmembrane potential and permeabilization

**Fig. 2:**
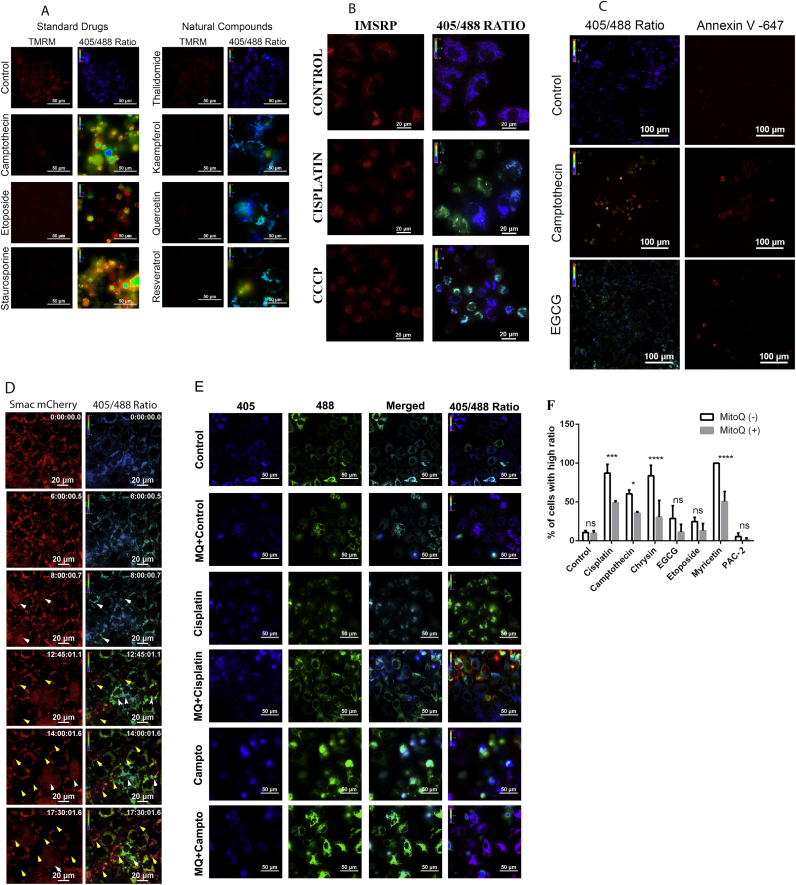
(A). U2OS cells stably expressing mt-roGFP were left untreated or treated with indicated drugs and natural compounds for 24 h. The cells were stained with TMRM to detect mitochondrial membrane potential as described. Control cells showed mitochondrial TMRM staining with an average ratio of 0.5 for roGFP. In treated cells, there is a significant drop in TMRM staining concomitant with an increase in the roGFP ratio. (B). U2OS cells stably expressing mt-roGFP were transiently transfected with IMS-RP plasmids. The cells were treated with cisplatin (150 µM), and CCCP (10 µM) for 24 h. Control cells showed mitochondrial red IMSRP fluorescence with an average ratio of 0.5 for roGFP. In treated cells, all cells with an increase in roGFP showed mitochondrial membrane permeabilization as evidence from diffused IMS-RP fluorescence. (C). U2OS cells stably expressing mt-roGFP were left untreated or treated with camptothecin (30 µM) and EGCG (50 µM) for 24 h. The cells were stained with annexin-V Alexa 647 to detect PS exposure to the plasma membrane as a measure of apoptosis. Control cells failed to show any annexin-V staining. In treated cells, there is a significant increase in annexin-V staining. (D). U2OS cells stably expressing mt-roGFP along with Smac-mCherry were treated with cisplatin (150 µM) and was subjected to fluorescent time-lapse imaging. The white arrowheads mark the cells’ initial surge in redox ratio concomitant with Smac-mCherry release. The yellow arrowheads indicate the drastic secondary increase in ratio subsequent to complete release of Smac-mCherry. (E). U2OS cells stably expressing mt-roGFP were either left untreated or pre-treated with MitoQ followed by drug treatment or drug alone as indicated for 24 h. Cells treated with MitoQ show lower redox ratio compared with corresponding drug alone treated. (F). Quantitative representation of the percentage of cell death in cells pre-treated with MitoQ followed by drug treatment or drug alone as indicated for 24 h.

Mitochondrial membrane potential and permeabilization are important markers of mitochondrial function. To understand whether the redox change in mitochondria is associated with its membrane potential loss, the cells stably expressing mt-roGFP were stained with TMRM. Upon drug treatment, cells with increased roGFP ratio also showed loss of TMRM indicating that both are dependent events. Moreover, cells with intact TMRM fluorescence retained low roGFP ratio (Fig. 2A).

Further to check the correlation of mitochondrial ROS and mitochondrial membrane permeabilization, the probe mCherry IMS-RP was introduced in mt-roGFP expressing cells. The IMS-RP probe is a non-functional variant of mitochondrial protein Smac - mCherry fusion protein that acts as an indicator of mitochondrial permeabilization in live cells [30]. As shown in Fig. 2B, cells with diffused IMS-RP red signal also showed increased roGFP ratio suggesting that redox alteration is always linked to cell death event and mitochondrial membrane permeabilization. Furthermore, analysis of the Annexin-V staining with redox changes showed that the cells with increased ratio also had increased Annexin-V positivity (Fig. 2C), confirming redox alteration is an accurate reflection of cell death. Intriguingly, in most of the drugs, we observed three to four-fold increase in the ratio in treated cells compared to control making this a promising approach for drug screening applications.

Despite the results described here showing a tight correlation between mitochondrial permeabilization and redox ratio increase at mitochondria, currently, it is not clear whether the oxidation of mitochondria is a cause or consequence of mitochondrial permeabilization. Dual-stable cells expressing both the intermembrane space protein Smac mCherry and mt-roGFP cells were developed and imaged in real-time mode to address this fundamental question in redox biology. The cells were treated with cisplatin and imaged continuously until their death. As seen from Fig. 2 D and video 1, prior to the release of Smac mCherry the cells showed an increase in the ratio from an average of 0.4 to 0.65; the ratio increased progressively to 1.2 with the release of Smac m cherry. Interestingly, after the complete release of Smac mCherry, mitochondria showed a further drastic increase in ratio. This suggests that the initial moderate increase in mitochondrial oxidation is a critical event in the mitochondrial permeabilization as detected by the ratio imaging.

Supplementary material related to this article can be found online at doi:10.1016/j.redox.2018.10.013.

The following is the Supplementary material related to this article Video 1.

Video 1 Time-lapse imaging cells stably expressing both mt-roGFP and Smac mCherry treated with cisplatin.

To confirm the essential role of mitochondrial oxidation in cell death, cells were pre-treated with the mitochondrial-targeted antioxidant MitoQ followed by ratiometric analysis of cell death. MitoQ pretreatment significantly decreased the mt-roGFP ratio change and cell death (Figs. 2E, 2F and Supplementary Fig. 4 and video 2). The results described here substantiate that mitochondrial oxidation is prior to mitochondrial permeabilization and this initial ROS alteration might contribute for the cell death through mitochondrial permeabilization.

Supplementary material related to this article can be found online at doi:10.1016/j.redox.2018.10.013.

The following is the Supplementary material related to this article Video 2.

Video 2 Time-lapse DIC image sequence of U2OS cells treated with Cisplatin alone in left panel and right panel shows cells treated with Cisplatin pre-treated with MitoQ.

### 3.3 High-throughput imaging and screening of drugs

**Fig. 3:**
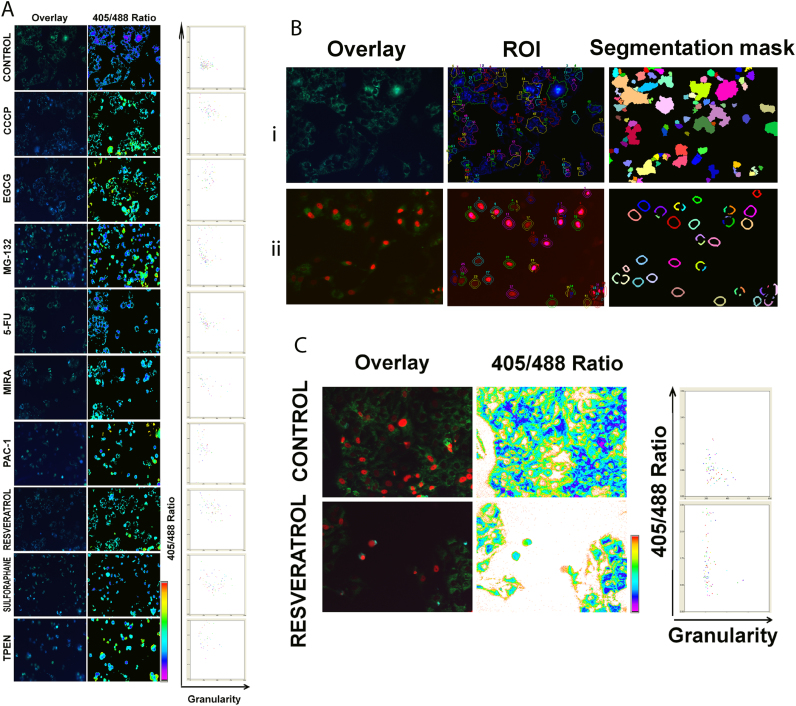
(A). U2OS cells stably expressing mt-roGFP were grown on 96-well imaging plates as described. The automated imaging was carried out for roGFP ratio. The merged image of 405 nm and 488 nm signal and ratio images from representative wells are shown. The scatter plot shows ratio against granularity of 488 nm signal. (B). (i) U2OS cells expressing mt-roGFP were segmented (upper panel) based on 405 nm intensity threshold, incomplete mitochondrial segmentation is evident. (ii) U2OS (lower panel) cells expressing mt-roGFP with H2B-mcherry were segmented based on mcherry intensity signal and polygon band as described. Complete and proper segmentation is evident. (C). U2OS cells expressing mt-roGFP with H2B-mcherry were treated with resveratrol (200 µM) for 24 h. Representative ratio image and scatter plot obtained from high-throughput image is shown.

The adaptability of the engineered cells for high-throughput screening was tested using a high-throughput Bioimager, BD Pathway 435. The bioimager was configured with required dual excitation filters for collecting single emission in ratio mode. The images were acquired after drug treatment. Post image acquisition, the cells were segmented based on the 405 nm signal. Representative images of untreated control and drug-treated wells are shown with respective scatter plots (Fig. 3A). The accuracy of the automated segmentation with 405 nm signal was not complete when cells are at higher density (Fig. 3B (i)). Stable introduction of the nuclear protein H2B-mCherry to these cells allowed a complete and accurate segmentation (Fig. 3B (ii)). The nucleus of all cells can be easily segmented, and the perinuclear defined polygon band acts as a readout plane in the ratio channel for quantification. A representative high-throughput image of untreated control and drug-treated wells with the representative quantitative ratio readout is shown in Fig. 3C. The calculated Z factor for the OVCAR8 cells without nuclear mCherry is 0.62 that has improved to 0.71 in cells expressing H2B-mCherry as a result of the accurate segmentation.

A panel of drugs with diverse chemical structure and function including natural products were screened. The representative ratio images and scatter plots are shown in Supplementary Fgure 5. As seen from the ratio images and scatter plots, the relative mitochondrial damage exerted by each compound is evident. Nigericin [34], the known inducer of inflammatory caspase 1 mediated cell death failed to induce any change in mt-roGFP ratio. The natural product EGCG also induced mitochondrial oxidation in a significant number of cells.

To track the time-dependent redox alterations and transmembrane potential loss at single-cell levels using automated imaging, TMRM loaded mt-roGFP cells were imaged at 2 h, 12 h and 48 h post-treatment. The same area was repeatedly imaged to capture the temporal changes at single cell level. The representative scatter plots showing roGFP ratio against TMRM intensity is presented in the supplementary Fig. 6. Compounds such as quercetin quickly dissipated TMRM fluorescence without considerable change in ratio (Supplementary Fig. 6). The ratio changes started later from 12 h and progressed among cells until 48 h. Remarkably, in a few drugs, the ratio increased up to five-fold at 48 h. The results presented here demonstrate and highlight that redox change is a better indicator of cell death than mitochondrial membrane potential loss, and it can be easily quantified.

### 3.4 Real-time imaging of redox alterations and transmembrane potential using wide-field and confocal imaging

**Fig. 4:**
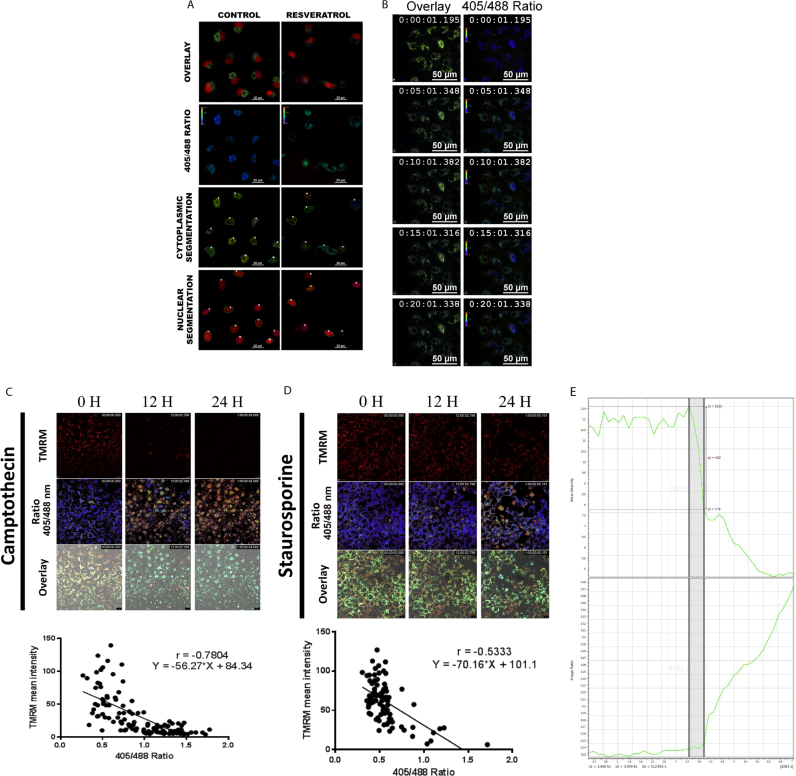
(A). U2OS cells expressing mt-roGFP with H2B-mcherry were segmented using Nikon NIS-elements software. (B). U2OS cells expressing Mito RO GFP were treated with resveratrol (200 µM), and real-time time-lapse imaging was carried out as described. Representative image at different time points is shown. (C). U2OS cells expressing mt-roGFP was stained with TMRM and treated with camptothecin (30 µM) for 24 h. The confocal imaging for ratio and TMRM was carried out as described. Representative ratio image, TMRM staining, and DIC images from indicated time points are shown. The lower panel shows the correlation plot of TMRM intensity with mt-roGFP ratio after 12 h of treatment. (D). U2OS cells expressing mt-roGFP was stained with TMRM and treated with staurosporine (2 µM) for 24 h. The confocal imaging for ratio and TMRM was carried out as described. Representative ratio image, TMRM image, and DIC images from indicated time points are shown. The lower panel shows the correlation plot of TMRM intensity with mt-roGFP ratio after 12 h of treatment. (E). U2OS cells expressing mt-roGFP was stained with TMRM and treated with camptothecin (30 µM) and for 24 h. The confocal imaging for ratio and TMRM was carried out as described. The TMRM release were plotted against roGFP ratio change.

Spatiotemporal changes of redox in the real-time mode were studied using both wide-field and confocal imaging. Representative automated segmentation of the cells in widefield microscopy is shown in Fig. 4A. Cells exposed to widefield imaging with proper illumination and exposure control allowed us to image the dynamics of mitochondrial ROS alterations and cell death induced by various cancer drugs for 24 h using a high sensitive EMCCD camera with an interval of 30 min. A representative image of cells treated with camptothecin is shown in Fig. 4B. The results substantiated that sub-spatial temporal changes occurring upon oxidation can be very well visualized using the approach at the single cell and subcellular levels.

Confocal imaging was performed to study the temporal alterations in redox changes and transmembrane potential loss induced by standard apoptosis-inducing agents with respect to control. In cells exposed to camptothecin (Fig. 4C and video 3), TMRM loss was immediately followed by an abrupt increase in mt-roGFP ratio and cell death. The result clearly shows a strong negative correlation between TMRM intensity and mt-roGFP ratio. The cells treated with kinase inhibitor staurosporine also followed a similar pattern (Fig. 4D, Video 4). However antioxidants such as EGCG and Resveratrol, ETC modulators such as CCCP and Valinomycin triggered early membrane potential loss but not immediately followed by redox changes or death (Supplementary Fig. 7 and video 6–9). The dose-dependent mt-roGFP ratio change at 24 h of CCCP and Valinomycin is shown in Supplementary Fig. 8. Detailed quantitative analysis revealed that transmembrane potential loss is the early event and thereafter redox alterations are initiated (Fig. 4E, video 5).

Supplementary material related to this article can be found online at doi:10.1016/j.redox.2018.10.013.

The following is the Supplementary material related to this article Video 3, Video 4, Video 5, Video 6, Video 7, Video 8, Video 9.

Video 3 Camptothecin: U2OS cells stably expressing mt-roGFP were stained with TMRM to detect Mitochondrial membrane potential loss as described. The cells were added with an indicated drug with 10 nm of TMRM. Live cell imaging was carried out as described.

Video 4 Staurosporine: U2OS cells stably expressing mt-roGFP were stained with TMRM to detect Mitochondrial membrane potential loss as described. The cells were added with an indicated drug with 10 nm of TMRM. Live cell imaging was carried out as described.

Video 5 U2OS cells stably expressing mt-roGFP stained withTMRM were treated with camptothecin and the ROI indicates the region from which the quantification data are derived as represented in figure 4E.

Video 6 Reserveratrol: U2OS cells stably expressing mt-roGFP were stained with TMRM to detect Mitochondrial membrane potential loss as described. The cells were added with an indicated drug with 10 nm of TMRM. Live cell imaging was carried out as described.

Video 7 EGCG: U2OS cells stably expressing mt-roGFP were stained with TMRM to detect Mitochondrial membrane potential loss as described. The cells were added with an indicated drug with 10 nm of TMRM. Live cell imaging was carried out as described.

Video 8 U2OS cells stably expressing mt-roGFP were stained with TMRM to detect Mitochondrial membrane potential loss. The cells were added with CCCP and Valinomycin respectively with 10 nm of TMRM. Live cell imaging was carried out for 2 h with an interval of 2 min

Video 9 U2OS cells stably expressing mt-roGFP were stained with TMRM to detect Mitochondrial membrane potential loss. The cells were added with CCCP and Valinomycin respectively with 10 nm of TMRM. Live cell imaging was carried out for 2 h with an interval of 2 min

### 3.5 Flow cytometry quantification

**Fig. 5:**
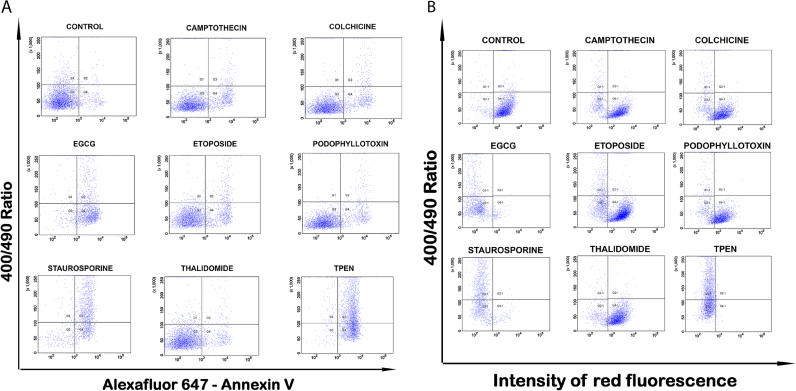
(A) U2OS cells stably expressing mt-roGFP were treated with different anticancer agents as indicated for 24 h. Cells were stained with Alexa 647- Annexin-V followed by FACS analysis of roGFP ratio against Annexin-V. (B). U2OS cells stably expressing mt-roGFP treated with different anticancer agents as indicated for 24 h after staining with TMRM as described. FACS analysis of roGFP against TMRM fluorescence was carried out.

For adapting the cell-based assay to flow cytometry, the single cell suspension prepared after indicated treatment were excited with 405 nm, and 488 nm laser lines and emission was collected at 520/40 nm from both laser line in ratio mode. Also, cells were stained with Annexin V for demonstrating the PS exposure. The cells with increased ratio also show increased Annexin V staining (Fig. 5A). In separate experiments, the roGFP ratio was analyzed with mitochondrial membrane potential using TMRM. This allowed simultaneous quantitation of two critical events of mitochondrial damage by flow cytometry using mt-roGFP cells. Most cells with increased ratio also showed reduced red fluorescence (Fig. 5B). Overall, the results described here demonstrate that the cell-based assay utilizing mt-roGFP cells can also be used for quantitative analysis of redox changes using flow cytometry and also for simultaneous imaging of other markers of cell death.

## 4 Discussion

The number of combinatorial chemistry-driven new compound libraries generated by pharmaceutical companies and academic organizations is increasing day by day. Proper identification of lead molecules with desired therapeutic activity requires extremely sensitive and high-throughput adaptable assays. For cancer drug screening, the most popular assays are MTT, Sulforhodamine and ATP based assays. Diverse apoptotic specific assays have also been described for cancer drug screening employing chemical probes that have led to the discovery of several promising leads. It has also been emphasized that best assays not only detect cytotoxic activity but also reflect the pathway of cell death signaling. Detection of redox changes within cells and their organelles is vital in understanding diseases and also drug screening and evaluation of environmental toxicants [35]. Even though chemical probes were extensively used for ROS detection, the probes such as dihydro-dichloro-fluorescein, dihydro-rhodamine are not specific for any particular oxidant, and imaging conditions very well influence their response [36]. However, MitoB, a boronate probe attached to a TPP+ moiety and MitoNeoD, a phenanthridinium based superoxide probe developed by Murphy and colleagues showed excellent sensitivity and selectivity as chemical probes of ROS [37], [38].

Genetically encoded probes have several advantages over chemical probes because they are specific and can be expressed in any cell or targeted to any organelle and enable real-time imaging possibilities. The best characterized genetically encoded redox sensors are HyPer and roGFP2-Orp1 for H2O2, roGFP1/2, rxYFP and roGFP2-Grx1 for GSH/GSSG redox state [25], [33], [39], [40].

In general, glutathione depletion is a typical hallmark of apoptosis, and an increasing number of studies suggest early GSH depletion promote the progression of cell death in response to diverse chemotherapeutics [41]. Changes in the intracellular thiol-disulfide (GSH/GSSG) balance are considered significant determinants of the redox status/signaling of the cell. It has been reported that oxidant injury at mitochondria is critical for the permeabilization, that is also associated with glutathione alteration [42]. The GSH/GSSG based roGFP shows dual excitation peaks at 400 nm representing neutral fluorophores and 490 nm representing the anionic form of the fluorophore. Oxidation-dependent disulfide formation promotes protonation of roGFPs leading to a fast increase of emission signal from 405 nm excitation and reduction on 488 nm excitation. This makes the probe a reliable indicator of redox potential once targeted at mitochondria for ratio imaging. Both roGFP and mt-roGFP were employed to understand the spatiotemporal changes in redox status in response to diverse physiological and pathological stimuli in cell lines and model organism [15]. Similarly, a microplate and microscopy-based approach to detect glutathione redox potential using Grx1-roGFP2 and H2O2 concentration using roGFP2-Orp1 were described by Dick TP and colleagues [43].

We have also employed a cytosolic roGFP to act as a cytosolic ROS sensor which gives a sharp fold increase in the ratio by flow cytometry. However, the assay fails to get promising *‘Z′* value in the high-throughput analysis because of the low 405 nm signal and high background (Data not shown). The focus of the work was to develop a sensitive image-based, redox-based drug screening tool. In addition the approach described here has potential applications in understanding fundamental redox questions because of its capability for multiplexing with other real-time signaling probes. Real-time simultaneous imaging of mitochondrial redox and mitochondrial permeabilization in cisplatin-treated cells reveals that before mitochondrial permeabilization, there is a detectable mito redox alterations that is followed by the progressive release of mitochondrial Smac. Also, the imaging confirmed a further rapid increase in ratio change after completion of release of Smac from mitochondria. This subsequent increase could be due to secondary necrosis of apoptotic cells. The above results in conjunction with inhibition of mitochondrial oxidation and cell death in mitoQ pretreated cells suggest that mitochondrial oxidation contributes for mitochondrial permeabilization.

The assay described here using roGFP targeted at mitochondria possess several advantages over other high-throughput assays based on conventional apoptosis detection methods. Compared to single intensity based fluorescent detection methods, the assay described here is ratiometric and is least influenced by compound fluorescence. Since the assay is live cell-based and there are no staining or washing methods, temporal analysis of cell death is very much possible. In addition, stably expressing single cell clones generated for the study retained the similar expression pattern even with repeated passaging and showed reproducible results indicating its robustness for drug screening. A major concern in using mt-roGFP probe to dissect cause and effect studies is its slow response to equilibrate with endogenous redox system. Eventhough, the mt-roGFP used in the current study (based on the roGFP2) is a fast responsive one than the original roGFP, the imaging results need to be carefully analyzed taking in to account its response time with precisely defined time-lapse experiments. As per the results shown here real-time imaging of CCCP and valinomycin treated cells with short intervals revealed complete dissipation of mitochondrial transmembrane potential before appreciable change in roGFP ratio.

An accurate segmentation using either 405 nm or 488 nm signal of mt-roGFP probe alone is not complete because of the inherent tendency of epithelial cells to grow as tight adherent cultures with minimum inter-cell margins. Introduction of a nuclear marker H2B-mCherry in these cells allowed easy and accurate segmentation. The *‘Z′* factor received with mt-roGFP only, and the improved *‘Z′* factor obtained with mcherry H2B expressing cells makes this a very promising high-throughput method for drug screening. We have developed and validated the suitability of this approach using six different cancer cell-lines from various tissue origin. All of them yielded similar performance for HTS. Currently, the laboratory is focusing on developing more cancer cell lines from the NCI panel so that a redox-based preliminary screening can be offered as an alternative easy to perform assay. One disadvantage of this approach is the difficulty in developing stable homogeneous expressing cells that require transfection, selection, and clonal expansion. However, once a stable cell line is developed, they will be a self-renewable resource, and subsequent cell death assay is easy to achieve cost-effectively with minimum assay variability. The most fundamental determinant of any successful HTS assay is its ability to detect the required biological readout in highly specific, reproducible, and inexpensive manner. Because of these unique advantages, this platform will be useful for profiling environmental toxic compounds in a rapid, cost-effective and systematic manner.

## 5 Conclusion

The current study describes a rapid, sensitive and easy to perform high-throughput assay for identifying compounds that induce mitochondrial damage and oxidation injury. The assay platform utilizes cancer cell-lines stably expressing redox-sensitive GFP targeted at mitochondria. The study confirms that oxidation injury to mitochondria as demonstrated with ratio imaging is a reliable marker for mitochondrial damage and outperform most apoptotic methods with good quantitative reliability. A significant advantage of this assay is the ease of use, versatility with any cell of choice, the possibility of multiplexing with other biological parameters and single cell as well as population level readout. The approach described here has promising applications in drug screening, toxicological evaluation of hazardous chemicals and also in dissecting signaling events relevant to redox biology.
