# Supplementary material for: A high-throughput real-time in vitro assay using mitochondrial targeted roGFP for screening of drugs targeting mitochondria
Source: Redox Biol. 2018 Oct 24;20:379–89. doi: 10.1016/j.redox.2018.10.013 (PMC6222140; doi:10.1016/j.redox.2018.10.013)
Supplement: Supplementary file 10 — Supplementary material [file mmc10.docx]

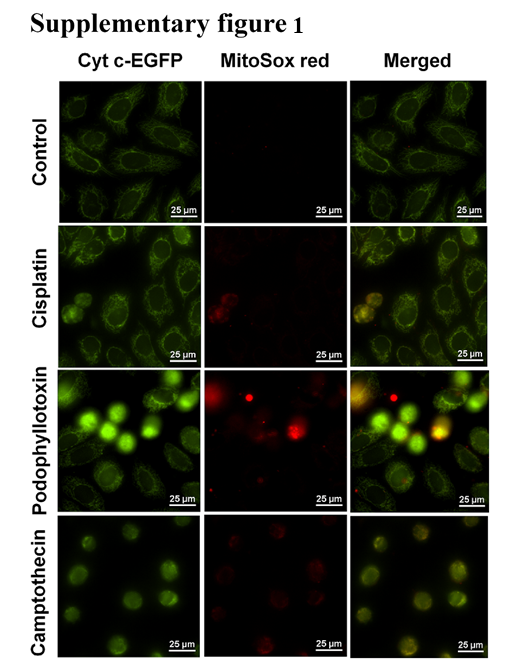


U2OS cells stably expressing Cytochrome *c*-EGFP were left untreated or treated with cisplatin (50µg/ml), podophyllotoxin (10µM) or camptothecin (10µg/ml) for 24 hours. The cells were stained with MitoSOX Red as described. Control cells showed negative staining for MitoSOX without indication for the release of cytochrome *c*-EGFP. In treated wells, all cells with diffuse EGFP showed an increase in MitoSOX (n=4).

U2OS cells stably expressing EGFP-Bax were left untreated or treated with cisplatin (50µg/ml), EGCG (50µM) or camptothecin (10µg/ml) for 24 hours. The cells were stained with MitoSOX Red as described. Control cells showed negative staining for MitoSOX without indication for the release of Cytochrome *c*-EGFP. In treated well, all the cells with granular EGFP Bax showed an increase in MitoSOX (n=4).


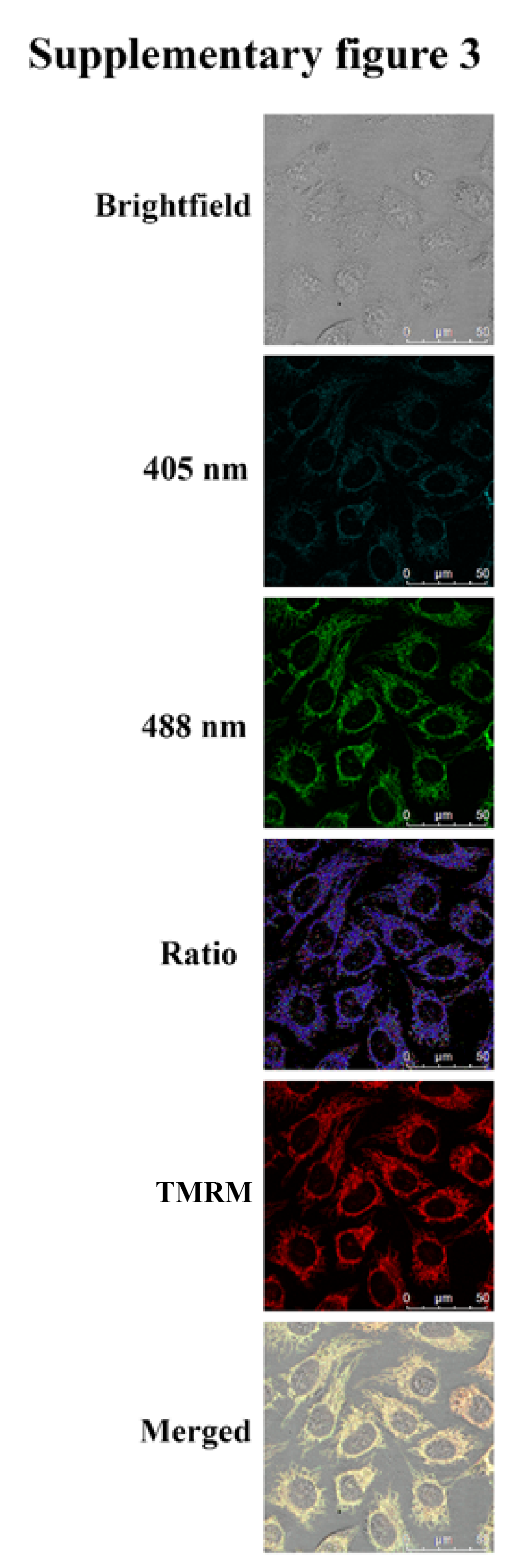


**Supplementary figure 3,** U2OS Cell stably and homogeneously expressing mt-roGFP. The mitochondrial targeting of mt-roGFP was confirmed by counter staining with TMRM dye.

**Supplementary figure 4**


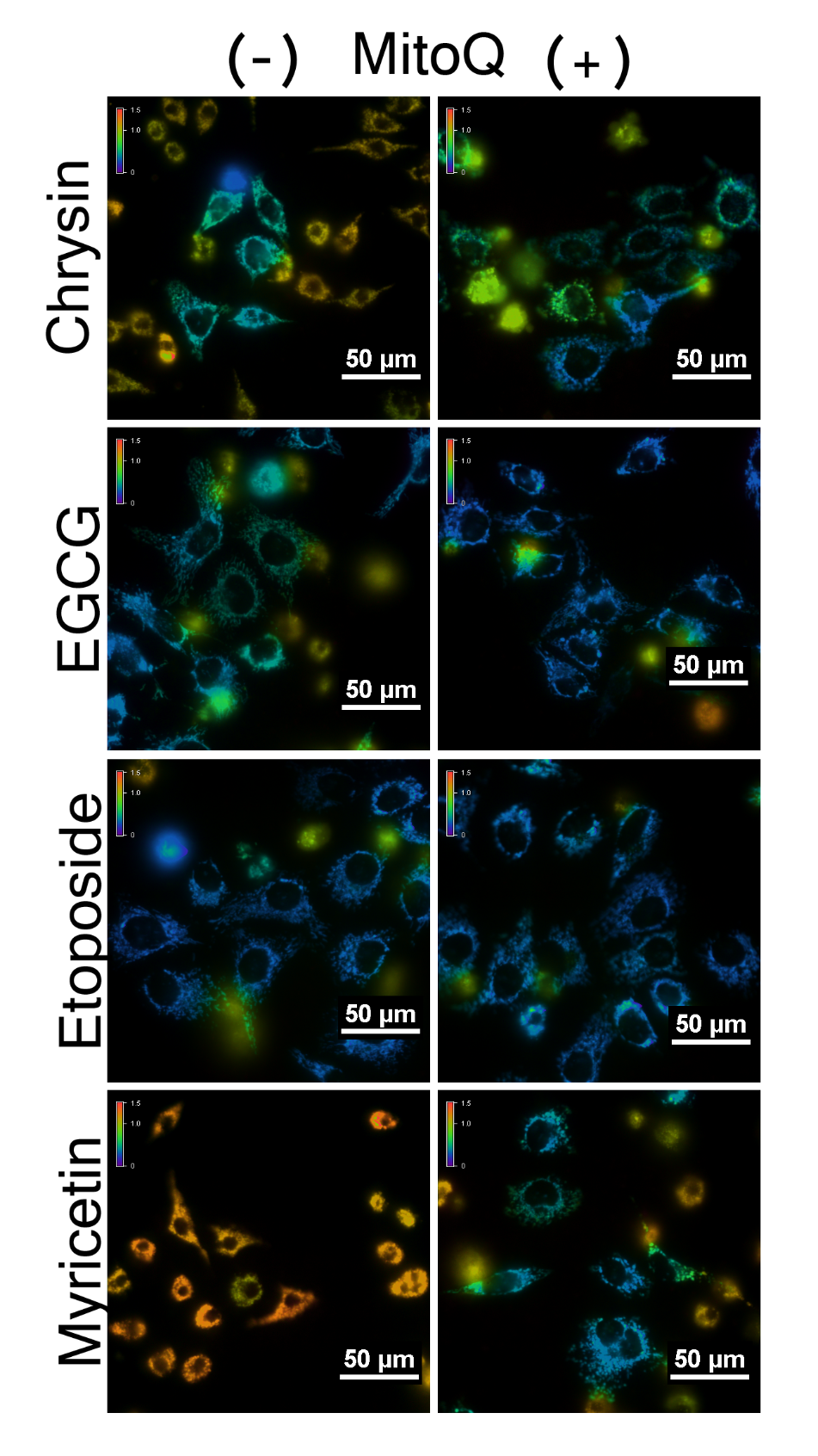


**Supplementary figure 4** U2OS cells stably expressing mt-roGFP were either left untreated or pre-treated with MitoQ followed by drug treatment (50µM) or drug alone as indicated for 24h. Cells treated with MitoQ show lower redox ratio compared with corresponding drug alone treated.

**Supplementary figure 5** U2OS cells stably expressing Mito roGFP were grown on 96-well plate imaging plates as described. The automated imaging was carried out for roGFP ratio. The merged image of 405 nm and 488 nm signal and ratio images from representative wells are shown. The scatter plot shows ratio against granularity of 488 nm signal.


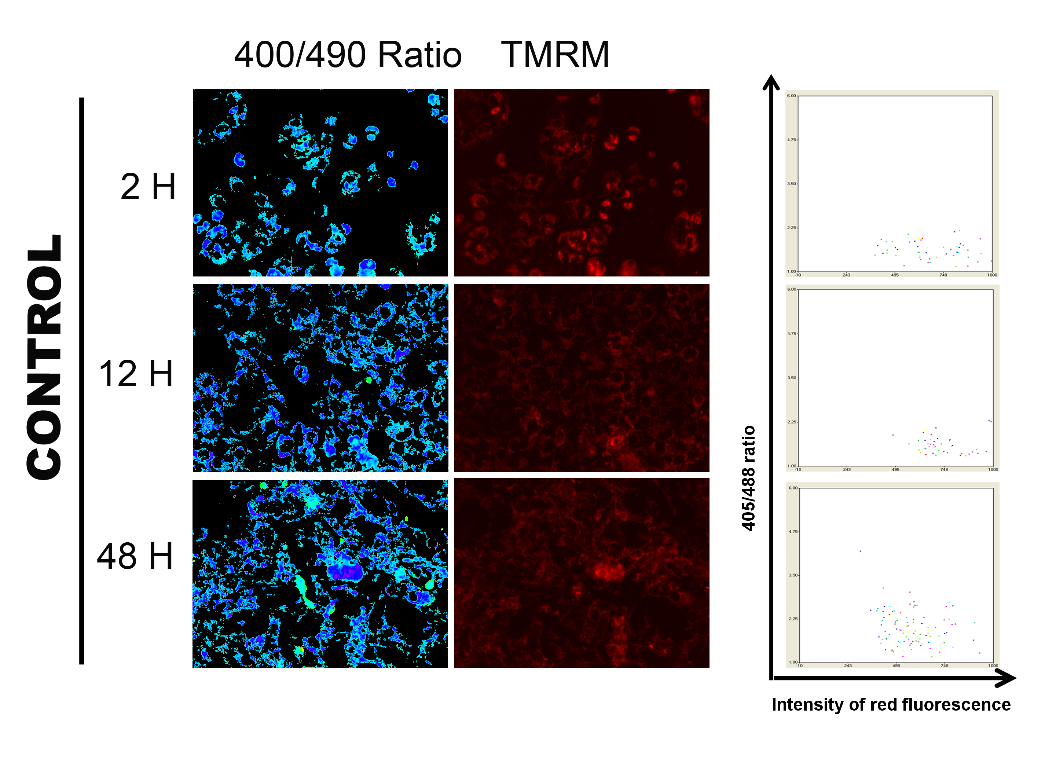

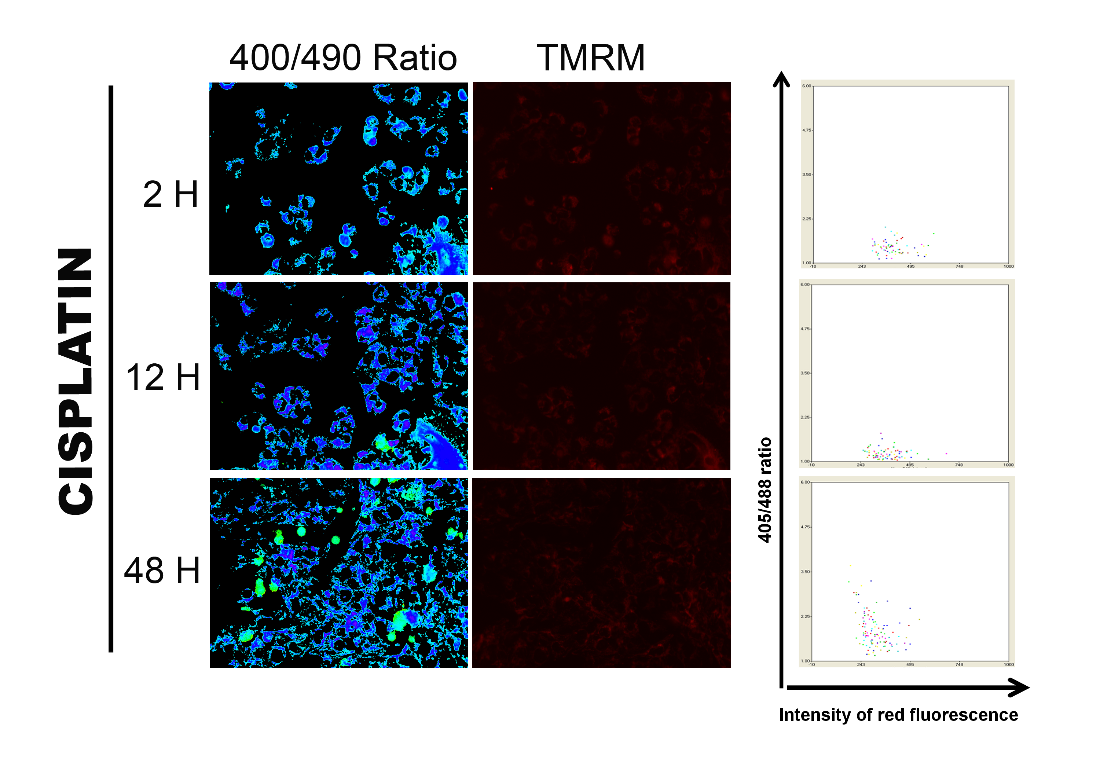

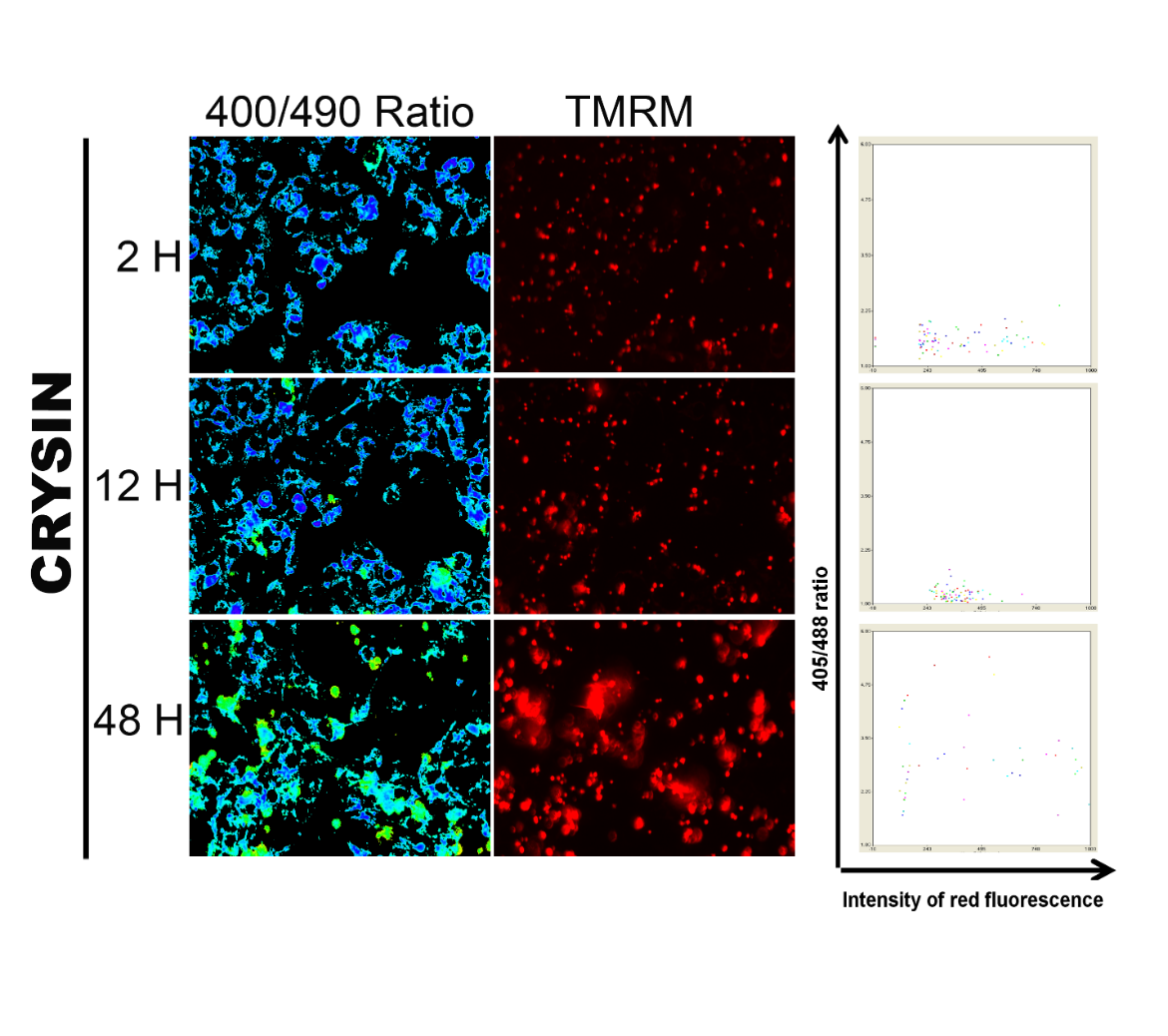

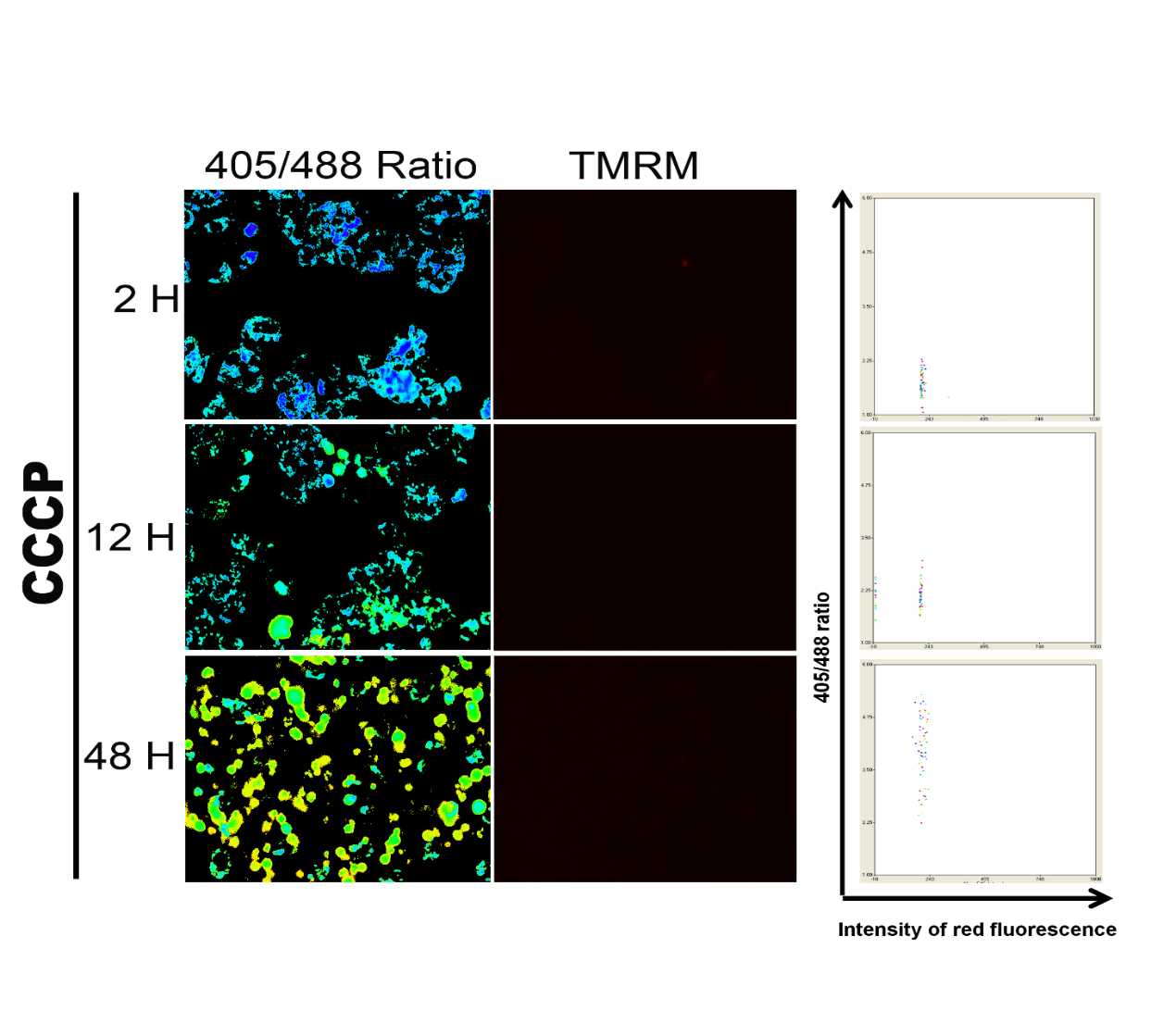


**Supplementary figure 6A**

**Supplementary figure 6B**


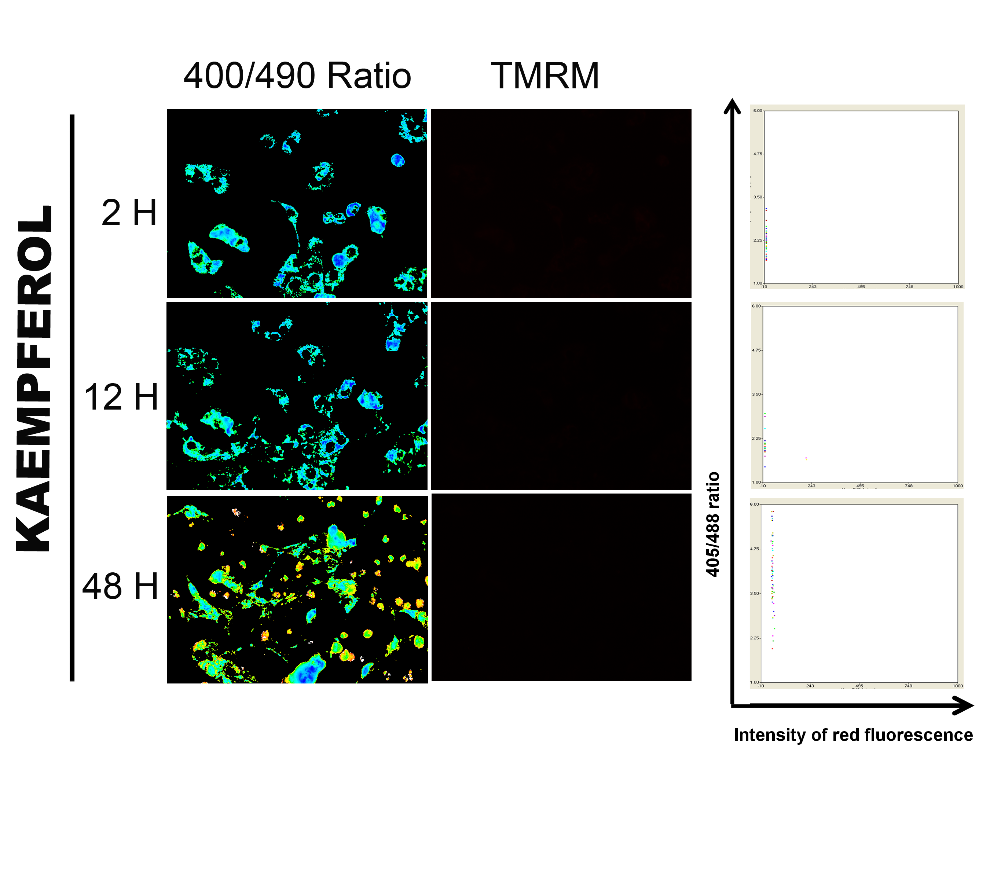

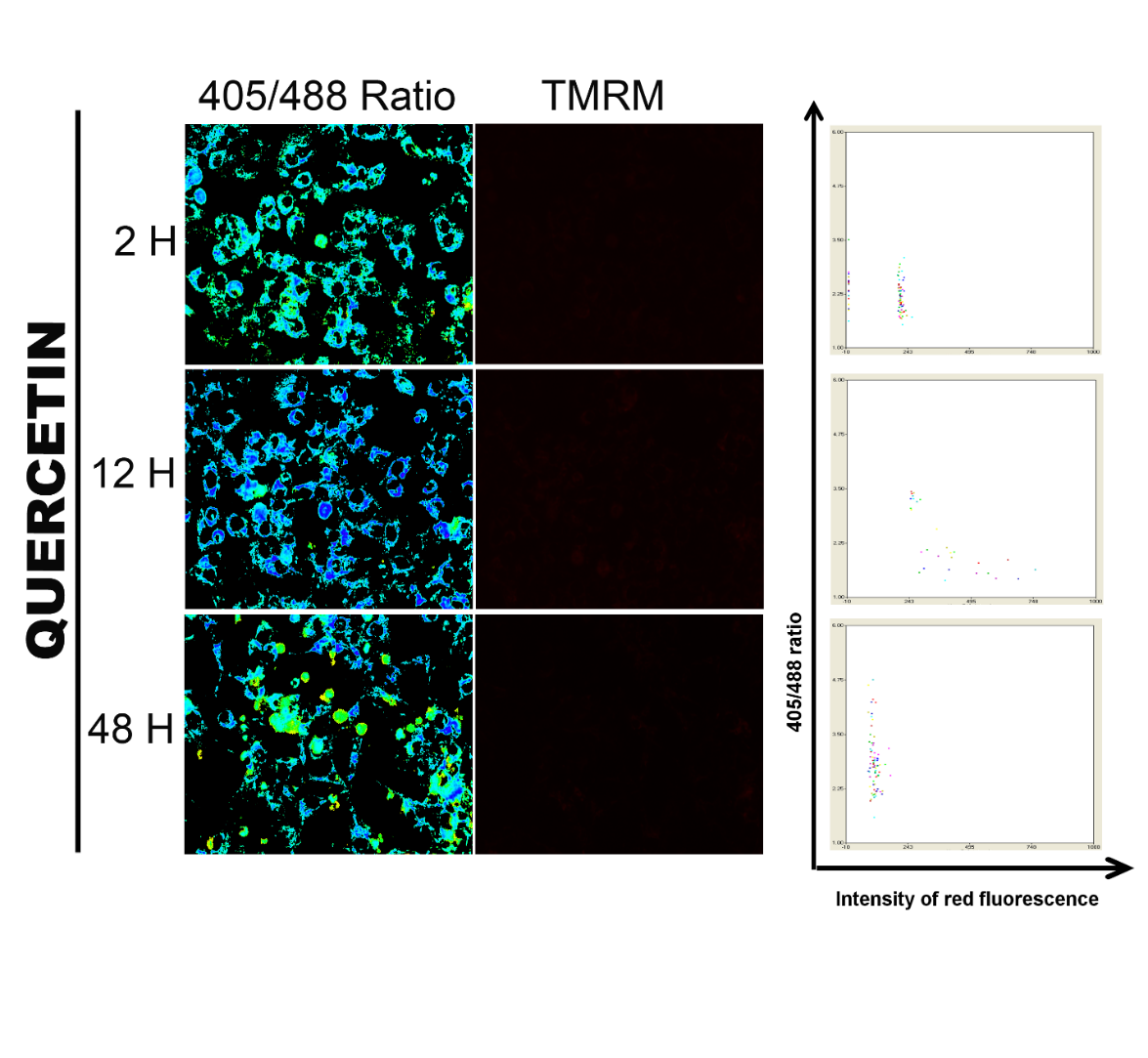


**Supplementary figure 6C**


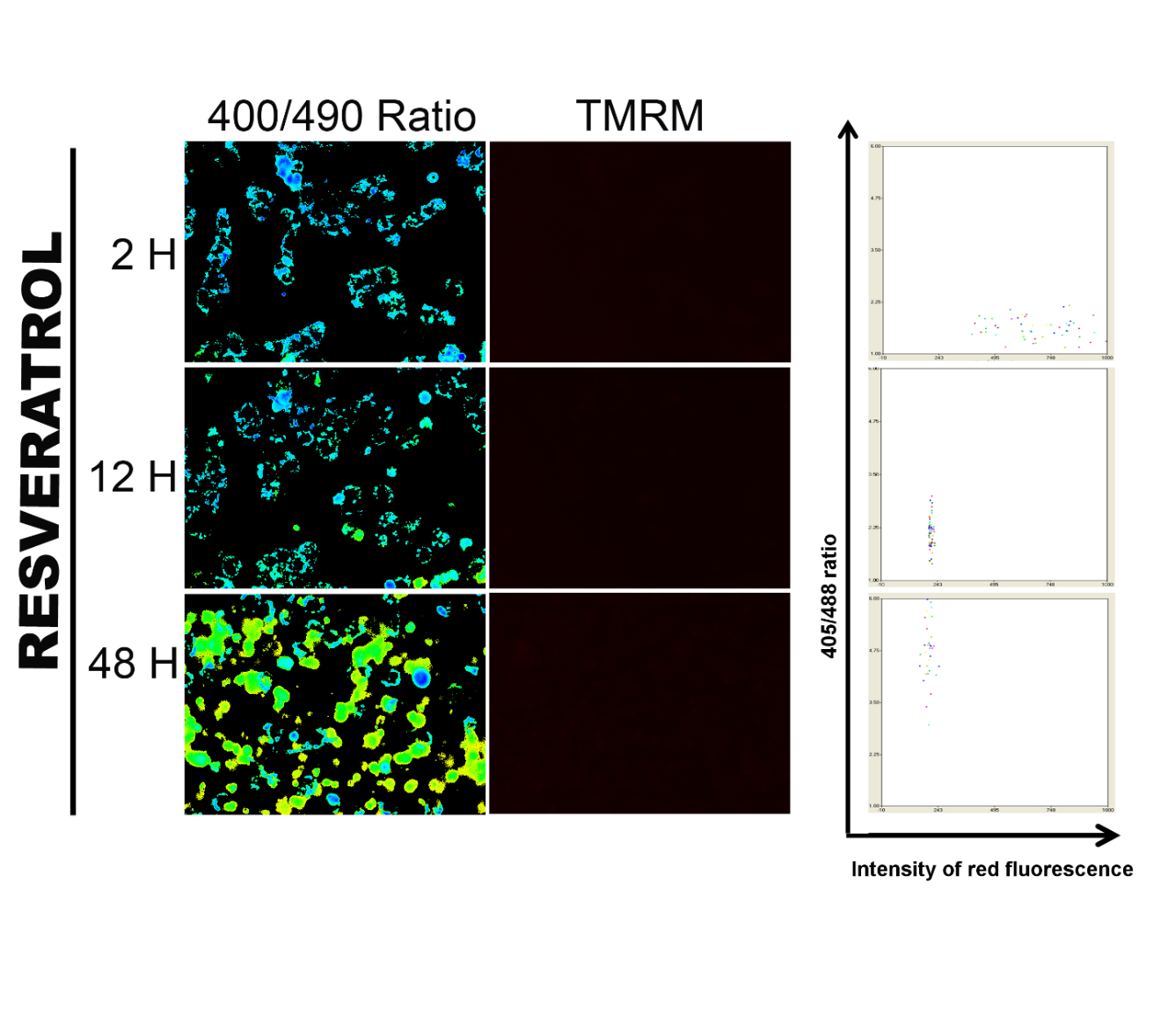

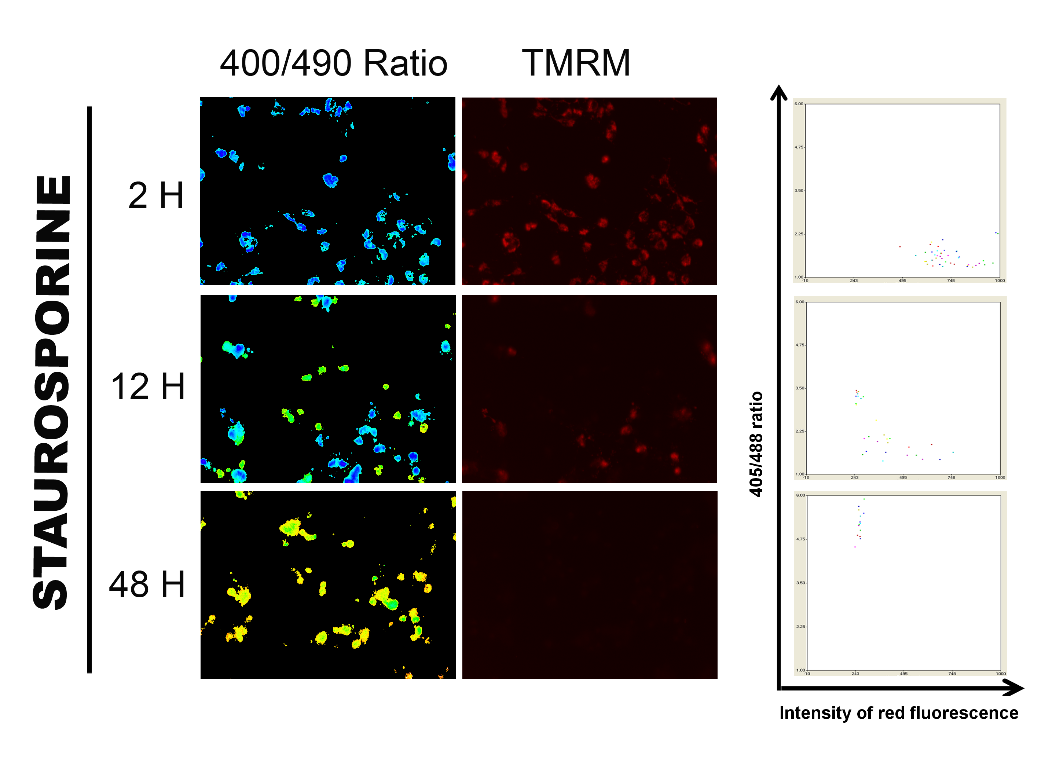


**Supplementary figure 6D**

**Supplementary figure 6A-D,** U2OS cells stably expressing mt-roGFP were stained with TMRM and treated with indicated drugs. Ratio images from representative wells are shown along with TMRM fluorescence. The scatter plot shows ratio against TMRM fluorescence.


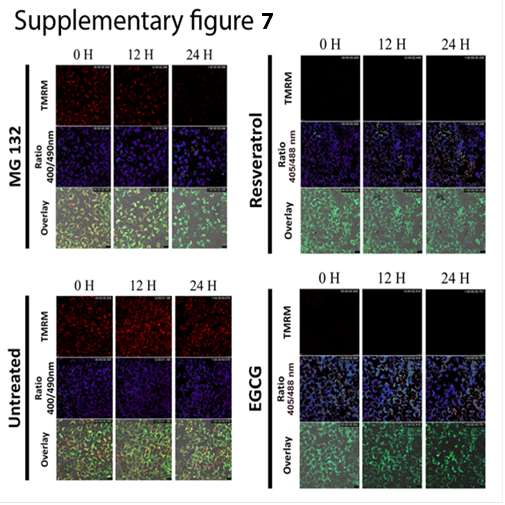


**Supplementary figure 7** U2OS cells stably expressing mt-roGFP were stained with TMRM and treated with indicated drugs. The merged image of 405 nm and 488 nm signal and ratio images from representative wells are shown along with TMRM fluorescence.


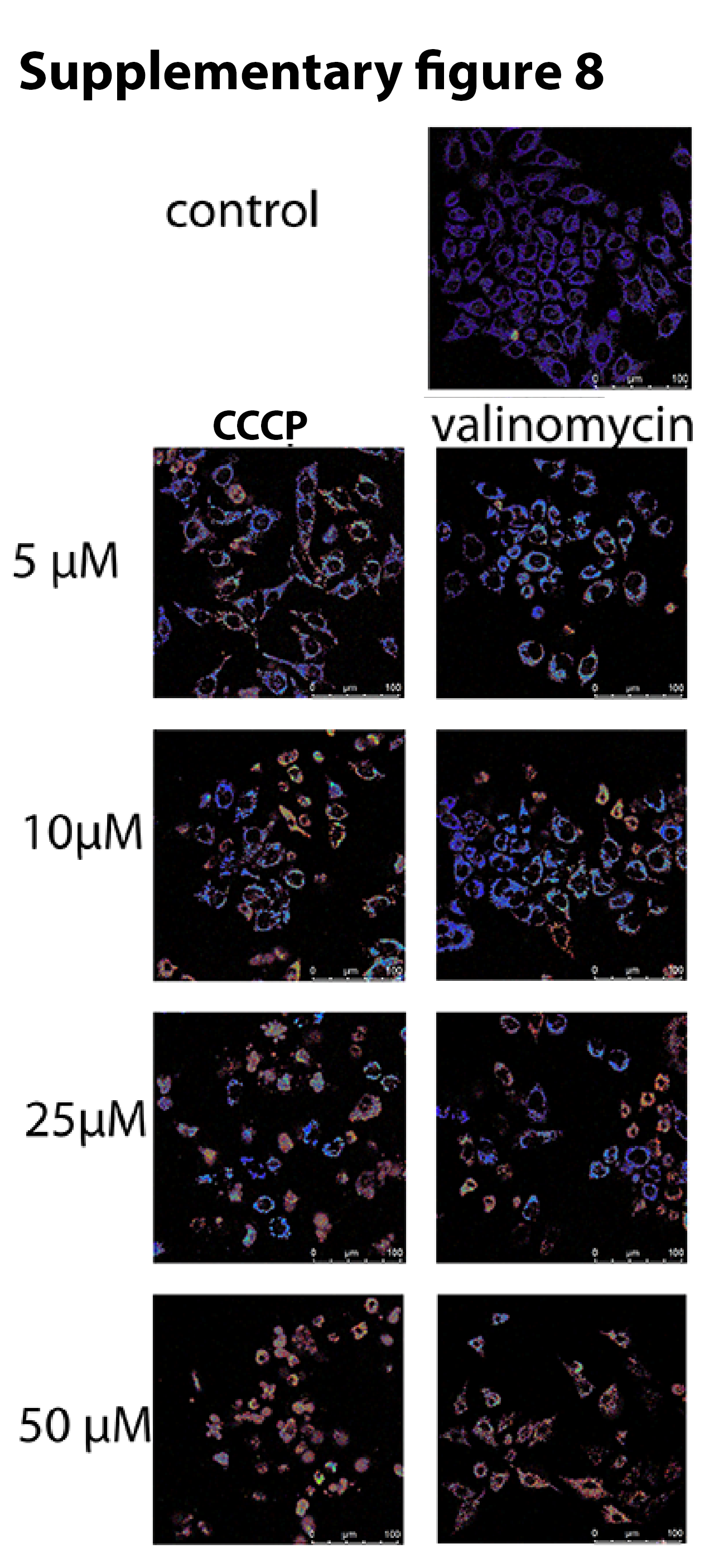


**Supplementary figure 8** U2OS cells stably expressing mt-roGFP were treated with increasing concentrations of indicated drugs and imaged ratiometrically.

Supplementary video 1 Time-lapse imaging cells stably expressing both mt-roGFP and Smac mCherry treated with cisplatin

Supplementary video 2: Time-lapse DIC image sequence of U2OS cells treated with Cisplatin alone in left panel and right panel shows cells treated with Cisplatin pre-treated with MitoQ. .

Supplementary video 5 : U2OS cells stably expressing mt-roGFP stained withTMRM were treated with camptothecin and the ROI indicates the region from which the quantification data are derived as represented in figure 4E

Supplementary videos : U2OS cells stably expressing mt-roGFP were stained with TMRM to detect Mitochondrial membrane potential loss as described. The cells were added with an indicated drug with 10 nm of TMRM. Live cell imaging was carried out as described.

3. Camptothecin

4. Staurosporine

6. Reserveratrol

7. EGCG

Supplementary video 8 and 9 : U2OS cells stably expressing mt-roGFP were stained with TMRM to detect Mitochondrial membrane potential loss. The cells were added with CCCP and Valinomycin respectively with 10 nm of TMRM. Live cell imaging was carried out for 2 hours with an interval of 2 min.
