# Supplementary material for: A high-throughput real-time in vitro assay using mitochondrial targeted roGFP for screening of drugs targeting mitochondria
Source: Redox Biol. 2018 Oct 24;20:379–89. doi: 10.1016/j.redox.2018.10.013 (PMC6222140; doi:10.1016/j.redox.2018.10.013)
Supplement: Supplementary file 11 — Supplementary material [file mmc11.docx]

# SUPPLEMENTARY TABLE 1

**Details of drugs used for screening in the study**

| **Sl. no.** | **Compound** | **Working concentration** |
| --- | --- | --- |
| **1** | Actinomycin D | 10 µM |
| **2** | Adenosine | 20 µM |
| **3** | 4-aminohydroxyflavone | 50 µM |
| **4** | Procaspase activator 1 | 25, 50 µM |
| **5** | Apoptosis Activator III (Embellin) | 50 µM |
| **6** | Procaspase activator 2 | 50 µM |
| **7** | Benzisoxazole | 5 µM |
| **8** | EGCG | 50 µM |
| **9** | Bleomycin | 200 µM |
| **10** | Caffeine | 50 µM |
| **11** | Chlorambucil | 200 µM |
| **12** | Chloramphenicol | 150 µM |
| **13** | Chrysin | 50 µM |
| **14** | Ciprofloxacine | 30 µM |
| **15** | Cisplatin | 150 µM |
| **16** | Cloxacillin | 10 µM |
| **17** | Colchicine | 125 µM |
| **18** | Curcumin | 25 µM |
| **19** | CCCP | 5, 10, 25 µM |
| **20** | Cycloheximide | 350 µM |
| **21** | Cyclophosphamide | 100 µM |
| **22** | Cytarabine | 4 µM |
| **23** | Dacarbazine | 1 mM |
| **24** | Docetaxel | 2 µM |
| **25** | Doxorubicin | 350 nM |
| **26** | Doxycycline | 11.25 µM |
| **27** | Etoposide | 25 µM |
| 28 | Nigericin | 1, 2 µM |
| **29** | Hydroxyurea | 2 µM |
| **30** | Hygromycin | 40 µM |
| **31** | Kaempferol | 100 µM |
| **32** | Kanamycin | 20 µM |
| **33** | Methotrexate | 5 µM |
| **34** | Mimosine | 200 µM |
| **35** | MIRA-1 | 10, 20 µM |
| **36** | Mitoxantrone | 1 µM |
| **37** | Gemcitabine | 50 µM |
| **38** | Rapamycin | 200 nM |
| **39** | Rutin | 200 µM |
| **40** | Sodium butyrate | 2 mM |
| **41** | Staurosporine | 1 µM |
| **42** | Taxol | 2 µM |
| **43** | Tetracycline | 100 µM |
| **44** | Thalidomide | 200 µM |
| **45** | Valinomycin | 5, 10, 25 µM |
| **46** | Zeocin | 68 µM |
| **47** | Thapsigargin | 2 µM |
| **48** | Zerumbone | 100 µM |
| **49** | Olomucine | 25 µM |
| **50** | Withaferin | 2 µM |
| **51** | Podophyllotoxin | 10 µM |
| **52** | Menandione | 20 µM |
| **53** | Resveratrol | 200 µM |
| **54** | Myricetin | 50 µM |
| **55** | Camptothecin | 30 µM |

**Supplementary Table S1:** List of the various drugs used for the screening for mitophagy inducers with the respective concentrations used.
